# [89Zr]-immuno-PET prediction of response to rituximab treatment in patients with therapy refractory interstitial pneumonitis: a phase 2 trial

**DOI:** 10.1007/s00259-023-06143-1

**Published:** 2023-02-24

**Authors:** H. Adams, E. M. W. van de Garde, D. J. Vugts, J. C. Grutters, Wim. J.G. Oyen, R. G. Keijsers

**Affiliations:** 1 Department of Nuclear Medicine, Groene Hart Hospital, Bleulandweg 10, 2803 HH Gouda, the Netherlands; 2 Department of Pulmonology, ILD Center of Excellence, St. Antonius Hospital, Nieuwegein, the Netherlands; 3 Department of Clinical Pharmacy, St Antonius Hospital, Nieuwegein, the Netherlands; 4 Division of Pharmacoepidemiology and Clinical Pharmacology, Utrecht University, Utrecht, the Netherlands; 5 Department of Radiology and Nuclear Medicine, Amsterdam UMC, Vrije Universiteit Amsterdam, Amsterdam, the Netherlands; 6 Division of Heart & Lung, University Medical Center Utrecht, Utrecht, the Netherlands; 7 Department of Biomedicals Sciences and Humanitas Clinical and Research Center, Department of Nuclear Medicine, Humanitas University, Milan, Italy; 8 Department of Radiology and Nuclear Medicine, Rijnstate Hospital, Arnhem, the Netherlands; 9 Department of Radiology and Nuclear Medicine, Radboud University Medical Center, Nijmegen, The Netherlands; 10 Department of Nuclear Medicine, St. Antonius Hospital, Nieuwegein, the Netherlands

**Keywords:** Interstitial lung disease, CD20, Rituximab, 89Zr-rituximab, Immuno PET, PET-CT

## Abstract

**Introduction:**

Immune-mediated interstitial pneumonitis may be treated with anti-CD20 therapy after failure of conventional therapies. However, clinical response is variable. It was hypothesized that autoreactive CD20-positive cells may play an important role in this variability. This prospective study aims to elucidate if imaging of CD20-positive cells in the lungs allows prediction of the response to anti-CD20 treatment.

**Methods:**

Twenty-one patients with immune-mediated interstitial lung disease (ILD) with deteriorated pulmonary function received a dose of 1000 mg rituximab on day 1 and day 14 spiked with a tracer dose of radiolabeled [89Zr]-rituximab. PET/CT was performed on days 3 and 6. Standardized uptake values (SUV) were calculated as a measure for pulmonary CD20 expression. Based on pulmonary function tests (PFT), forced vital capacity (FVC), and diffusing capacity for carbon monoxide (DLCO), prior to and 6 months after treatment, patients were classified as responder (stable disease or improvement) or non-responder.

**Results:**

Fifteen patients (71%) were classified as responder. Pulmonary [89Zr]-rituximab PET SUVmean was significantly correlated with the change in FVC and DLCO (*K* = 0.49 and 0.56, respectively) when using target-to-background ratios, but not when using SUVmean alone. [89Zr]-rituximab SUVmean was significantly higher in responders than in non-responders (0.35 SD 0.09 vs. 0.23 SD 0.06; *P* = 0.02).

**Conclusion:**

Rituximab treatment was effective in the majority of patients. As a higher pulmonary uptake of [89Zr]-rituximab correlated with improvement of PFT and treatment outcome, [89Zr]-rituximab PET imaging may serve as a potential predictive biomarker for anti-CD20 therapy.

**Trial registration:**

Clinicaltrials.gov identifier NCT02251964

**Supplementary Information:**

The online version contains supplementary material available at 10.1007/s00259-023-06143-1.

## Introduction

While novel immunotherapeutic strategies play an important role in oncological and rheumatological diseases, their role in rare immune-mediated inflammatory diseases (IMIDs) with interstitial pneumonitis (IP) has only recently been addressed [1, 2]. On the road to personalized medicine, there is a need to identify predictive biomarkers that can help select patients for immunotherapy by identifying potential (non-)responders. Despite the lack of large prospective studies, the urgent need for clinically necessary treatment, especially in life-threatening situations, sometimes makes the off-label use of certain immunotherapies in rare lung diseases unavoidable. Rituximab is one of the most prominent therapeutic antibodies with a comprehensive safety and efficacy record. The efficacy of rituximab therapy in rheumatoid arthritis and granulomatosis with polyangiitis, which are closely related, is well documented [3]. Rituximab targets CD20 receptors on B cells with high specificity, leading to a depletion of CD20 cells from the blood pool and a subsequent decrease in systemic inflammation [4]. The presence of CD20+ B cells in immune-mediated interstitial pneumonitis (IMID-IP) has been well documented and confirmed in several studies [5–9]. However, response to treatment with rituximab varies considerably in IMID-IP and effective biomarkers for patient stratification are lacking. Current literature reports response rates between 30 and 60%, especially when measuring forced vital capacity (FVC) [2, 10]. The variable improvement in lung function after rituximab therapy could be the result of different pathophysiological mechanisms, such as repopulation of CD24+/CD34+ regulatory B cells by restoring IL-10 production and normalization of invariant natural killer T cell function [9]. However, a possible relationship between the number of CD20 targets in the lung and the response to rituximab has not yet been investigated. If a non-invasive biomarker could measure the presence of CD20 cells in the lungs, this would potentially be of use to distinguish clinical responders from non-responders before treatment is started. Since most patients with IMID-IP cannot safely undergo invasive procedures due to the high risk of biopsy-related complications, a non-invasive biomarker is needed to safely assess the presence of CD20 cells. We hypothesized that the amount of pulmonary CD20 targets is directly related to response to rituximab. Previously, we demonstrated the safety and potential use of [89Zr]-rituximab immune PET /CT as a non-invasive specific biomarker for CD20 cells in a feasibility study (*N* = 10) [11]. This is the first prospective trial to investigate the predictive potential of this novel imaging technique in IMID-IP and to compare imaging data with clinical outcome after rituximab treatment.

## Methods

**Fig. 1:**
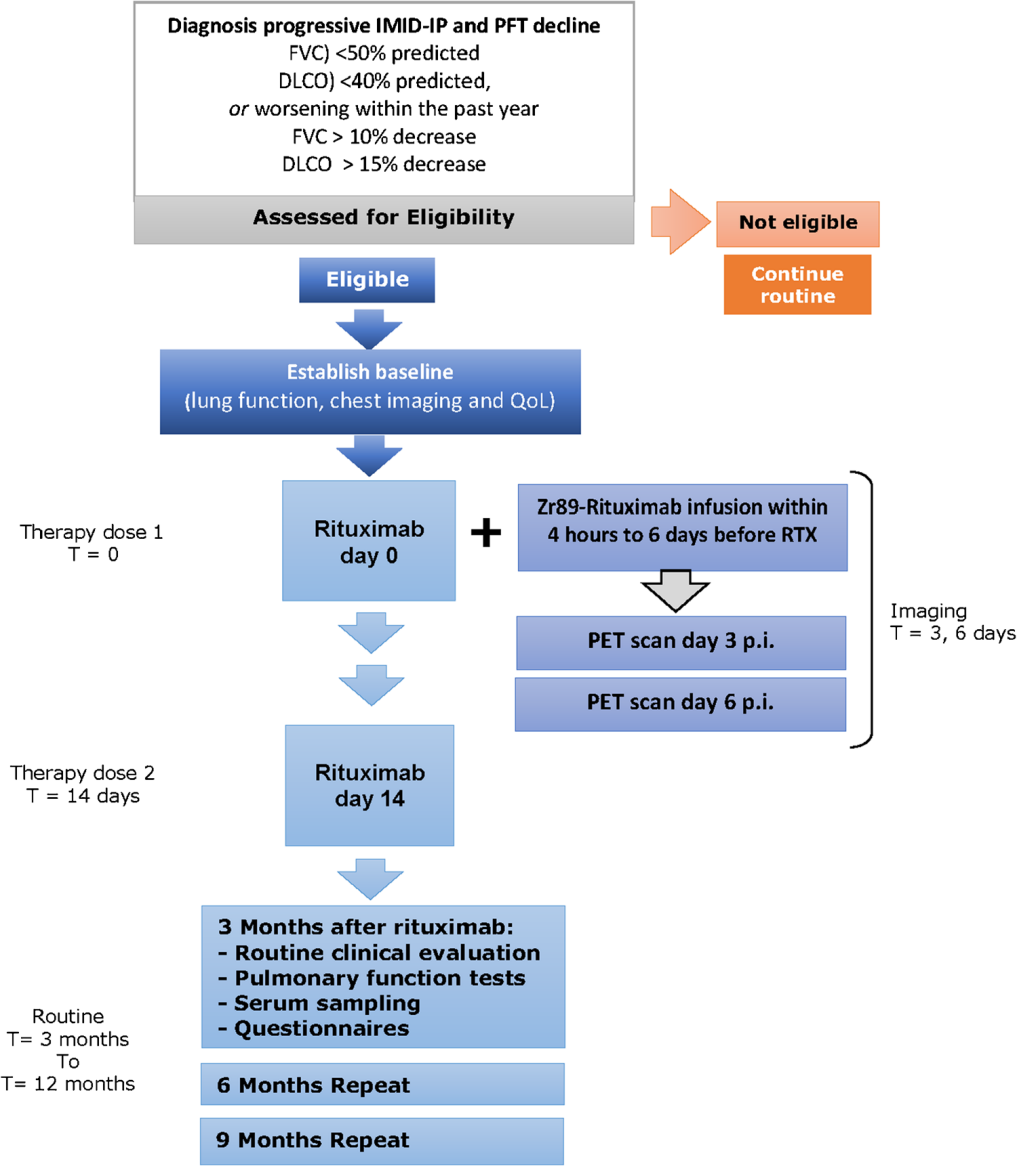
Our study protocol. Patients were selected based on a diagnosis of IMID and ILD and their eligibility for intervention according to the study criteria (see corresponding sections). Patients were enrolled in follow-up as soon as they completed rituximab therapy doses 1 and 2. At least one imaging session on day 3 was required for inclusion in the imaging analysis

This study was conducted to further evaluate the therapeutic efficacy of rituximab in a Dutch cohort of patients with IMID-IP in a prospective phase II study. Patients were referred to the Department of Pulmonology at St. Antonius Hospital Nieuwegein, a national center for the treatment of rare lung diseases, between 2015 and 2017. The study design was approved by the Medical Research Ethics Committee (MEC-U) under NL49534.100.14. This study is registered at ClinicalTrials.gov under the identifier NCT02251964. All patients provided written informed consent prior to participation. An overview of the study interventions is summarized in Fig. 1.

### Inclusion criteria

Treatment-refractory patients with progressive IMID-IP and without prior immunotherapy were eligible for participation if the following inclusion criteria were met: diagnosis of IMID and ILD by a specialized multidisciplinary team consisting of ILD pulmonologists, thoracic radiologists, rheumatologists, and pathologists. Diagnosis of IP progression by a multidisciplinary team guided by the strict criteria of the study protocol: (I) at least two pulmonary function tests within the last 6–9 months; (II) pulmonary function: forced vital capacity (FVC) less than 50% predicted and/or diffusing capacity for carbon monoxide (DLCO) less than 40% predicted or deterioration in pulmonary function demonstrated by any of the following within the last year (more than 10% decrease in FVC, more than 15% decrease in DLCO). In addition, patients were considered refractory to first-line therapy (corticosteroids) and second-line therapy (cyclophosphamide or azathioprine). Exclusion criteria were residual lung volume greater than 120% of the predicted value at screening and DLCO less than 25% of the predicted value at screening, measured at rest. Patients who showed signs of infection were excluded. The detailed criteria for the study, registered as rituximab in interstitial pneumonitis (RITUX-IP), can be found in the supplementary file (Table S1). The study can also be found online under the identifier NCT02251964 2018 at ClinicalTrials.gov.

### Intervention

All patients who met the inclusion criteria were to receive a therapeutic dose of rituximab as an adjunct to their immunosuppressive treatment. One thousand milligram of rituximab was administered intravenously on day 1 and day 14, after premedication with paracetamol, dexamethasone, and antihistamines according to protocol. Maintenance therapy with low-dose steroids (5 to 10 mg prednisone) remained unchanged in the rituximab regimen, as did maintenance therapy with immunosuppressive drugs (either azathioprine and or cyclophosphamide) and, in one case, low-dose methotrexate. After rituximab therapy, immunosuppressive medication was not increased. Adverse and serious adverse events were documented.

### Pulmonary function tests

PFTs were performed within 2 weeks before day 1 of rituximab therapy and repeated 6 months after day 14 of rituximab therapy. Predicted diffusing capacity of the lung for carbon monoxide (DLCO) and FVC was measured using a MS-PFT analyzer (Jaeger, Wuerzburg, Germany) in accordance with European Respiratory Society guidelines [12]. The percentages of predicted FVC and DLCO were used for data analysis. Subsequent PFT values with a decrease in FVC of more than 10% and/or DLCO of more than 15% were considered clinically significant decreases, whereas an increase in FVC of 10% and/or DLCO of 15% or more was considered a clinically significant improvement. PFT values that changed by less than 10% in FVC and less than 15% in DLCO were considered clinically stable values.

### Biomarker measurements

Blood samples were collected for biomarker analysis before infusion of rituximab. B cell depletion, indicative of the effect of rituximab treatment, was measured at baseline and post-therapy using CD19 rather than CD20 to avoid measurement errors masked by rituximab. Systemic antibodies such as antinuclear antibodies (ANA) and anti-neutrophil cytoplasmic antibodies (ANCA), usually associated with IMID, were measured to determine autoimmune parameters. For inflammatory lung parameters, soluble interleukin-2 receptor (sIL-2R), procalcitonin (PCT), IL-18, and CA 15.3 were used. The CD4:CD8 ratio and chemokine ligand 18 (CCL18) were also measured before and after treatment with rituximab.

### Chest imaging

Chest radiographs and high-resolution computed tomography of the chest (HRCT) were routinely performed to assess disease response according to local guidelines (thin-section images CT of 1–1.5 mm with a high spatial frequency reconstruction algorithm at 120 kV and approximately 240 mAs). All HRCT scans were evaluated by a specialist thoracic radiologist. Discrepant interpretations were discussed in the multidisciplinary team meeting until consensus was reached.

### Criteria of clinical response assessment

Patients were assessed for health status (quality of life or QoL), blood samples, pulmonary function parameters (FVC, DLCO), and serial imaging HRCT at two time points, at baseline and after 6 months (+/− 3 weeks). Two independent pulmonologists, specialized in ILD, independently evaluated the aforementioned parameters and assessed the treatment response. Patients were categorized into three types of clinical response: non-responders (NR; worsening of clinical condition), stable responders (RSP_stable_), and improving responders (RSP_improv_). Consensus was reached when both pulmonologists came to the same conclusion about the type of response.

### [89Zr]-rituximab PET/CT

Patients received an intravenous dose of 10 mg of the targeted anti-CD20 agent rituximab, radiolabeled with 18 MBq of ^89^Zr ([89Zr]-*N*-suc-DFO-rituximab, abbreviated as [89Zr]-rituximab) [13, 14]. ^89^Zr (half-life 3.27 days) immunoPET requires a distinctly different protocol than standard ^18^F tracers, as several factors may impact the scan results, such as the specific order of administration of radiopharmaceutical and therapeutic rituximab. The first 10 patients received substances in the following order: therapeutic rituximab is administered first, followed by [89Zr]-rituximab within 4 h later. In the remaining patients, the order is reversed: [89Zr]-rituximab is administered first, followed 6 days later by therapeutic rituximab. The reversal of the order is for signal-to-noise ratio analysis in the images from PET, which was approved by MEC-U. Images were obtained using a Philips Gemini TF PET/CT (Philips Medical Systems, Best, The Netherlands) adapted for ^89^Zr imaging according to the Zr-harmonization protocol [14]. Acquisition time of [89Zr]-rituximab PET images was 15 min per bed position from the lower neck to the splenic region. Imaging time points were 3 and 6 days after injection of [89Zr]-rituximab. All patients will receive two scans.

### Image analysis of [89Zr]-rituximab PET/CT

The data was normalized and corrected for several factors including decay before reconstruction to account for time elapsed between injection and scanning (1–2 half-lives). A post-reconstruction Gaussian filter was applied as recommended for [89Zr] immuno-PET studies [14, 15]. The lung, liver, and blood pool (measured as mean activity in the left ventricle) were evaluated using predefined volumes of interest (VOIs) of 4–14 cm^3^ (1–1.5 cm radius). To reduce interobserver variability, we used semi-automated lung segmentation software (Hermes Medical Solutions, Stockholm, Sweden) to obtain VOI measurements of the entire lung parenchyma. These VOI measurements of the lung were then used to calculate standardized uptake values (SUV) data of the lung (SUVmean of the lung and SUVmax). Additional target-to-background ratios were calculated by dividing SUVmax by blood pool SUV.

### Statistics

#### Estimating sample size

Assuming that lung function cannot improve spontaneously, it was calculated that a sample size of 18 subjects is sufficient to detect a statistically significant difference from baseline lung function using G*Power 3.1.7 software [16]. For this calculation, input data was used from other studies [17–19]. These reported an average improvement in lung function of 35.5%. To account for 10% of participants lost to follow-up, the total sample size was set at a minimum of 20 participants.

#### Data statistics

Independent and paired *T* tests were performed for continuous variables with normal distribution. Correlations between biomarkers and outcome measures were performed using bivariate correlation with Pearson’s correlation coefficient and a two-sided significance test. Differences in median PFT before and after rituximab treatment were assessed using the median test for independent samples, and interquartile range (IQR) is presented in parenthesis following the median. Statistical analysis was performed with SPSS version 22 (IBM, Armonk, NY, USA).

## Results

### Patient demographics

Informed consent was obtained from 23 patients. Subsequently, 21 patients received rituximab therapy and CD20 imaging. The remaining 2 patients could not participate in CD20 imaging because of the unavailability of [89Zr]-rituximab and were excluded. Patient characteristics are summarized in Table 1. Supplementary Table S1 lists all patient characteristics in detail. Four patients (19%) had rheumatoid arthritis (RA)-associated interstitial lung disease (ILD), six patients (28.6%) had antisynthetase syndrome (ASS) related IP, seven (33.3%) patients had hypersensitivity pneumonitis (HP), and four patients (19%) had other types of connective tissue disease-related IP (CTD-ILD). The former smokers (33.3%) had an average of 25.5 pack-years. At baseline, patients had a relative FVC drop of − 9.5% and a relative DLCO drop of − 15.9% in the 6 months before study participation. All patients had severely decreased FVC and/or DLCO; mean FVC at baseline was 67.6% (percent predicted) and 35.2% DLCO (percent predicted). Systemic antibodies such as ANA and ANCA were present in a minority of patients (19% and 9.5%, respectively). sIL-2R was highly elevated in all patients at baseline. On the basis of HRCT, 10 patients (47.6%) had non-specific interstitial pneumonia (NSIP) (of which seven had fibrotic NSIP and two had organizing pneumonia), four (19%) had ordinary interstitial pneumonia (UIP), and seven (33.3%) had hypersensitivity pneumonitis (HP).

**Table 1:** Baseline characteristics of patient’s clinical and functional data (*N =* 21)

	Mean SD (*n*)
Age (years)	60.3 SD 10.2 (*N =* 21)
Never smoker Previous smoker Pack-years (years)	66.7% (*N =* 14) 33.3% (*N =* 7) 25.5 SD 10.9
Relative FVC decline (%) past 6–9 months prior to study	− 9.5 SD 5.9
Relative DLCO decline (%) past 6–9 months prior to study	− 15.9 SD 14.5
Baseline FVC (% predicted)	67.6 SD 17.4
Baseline DLCO (% predicted)	35.2 SD 8.7
ANA positivity *(cut-off ≥ 1:160*	19% (*N =* 4)
ANCA positivity *(cut-off ≥ 1:160*	9.5% (*N* = 2)
sIL2R (reference < 3000 pg/ml)	4829 SD 2710

### Adverse events and serious adverse events

There were three serious adverse events where patients presented progressive dyspnea, requiring admission at the emergency department. Of these, one patient experienced further disease progression leading to death approximately 1 month after therapy. One patient developed a severe anaphylactic allergic reaction after the first 100 mg infusion of rituximab, resulting in discontinuation of rituximab treatment. One patient presented with trauma and subsequent humerus fracture, unrelated to rituximab therapy.

### Treatment response

There were no discrepancies in response type assessment between the two independent pulmonologists, so consensus was reached independently in all patients (details can be found in the supplementary Table S3). Overall, 71.4% (*N* = 15) of patients were classified as responders to rituximab therapy, nine patients were stable responders (RSP_stable_), and six patients were improving responders (RSP_improv_). The RSP_stable_ group showed a mean change in FVC and DLCO of + 3.6% and − 0.34%, while the RSP_improv_ group showed a mean change of + 14.8% and + 8.1%, respectively. In RSP_improv_, all PFT values improved. Six patients were classified by consensus as NR (mean decrease in FVC and DLCO of − 9.8% and − 0.4%, respectively). There were 2 patients in the NR group who initially showed stable PFT values after rituximab treatment but deteriorated in physical condition and showed increase of ILD on HRCT.

**Fig. 2:**
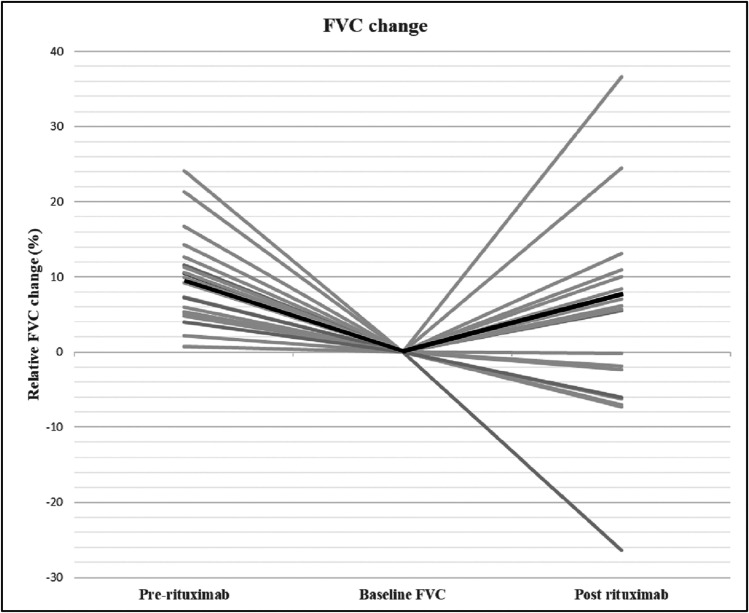
Relative FVC change (%) 6 to 9 months prior to rituximab therapy and 6 months after (*N =* 21). A median decline of 9.3% (− 5.3 to − 12.1) was found prior to rituximab and a median improvement of 5.7% (9.2 to − 4.2) after rituximab therapy (*P =* 0.002)

**Fig. 3:**
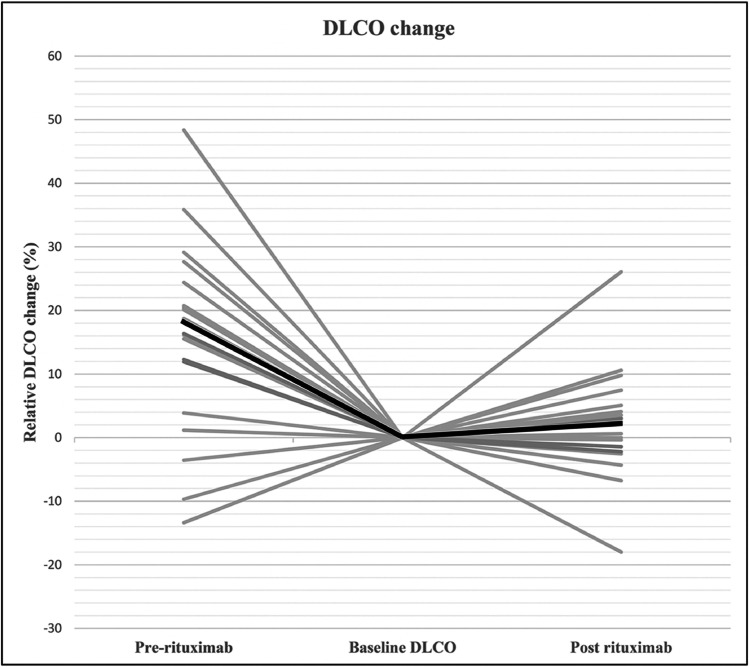
Relative DLCO change (%) 6 to 9 months prior to rituximab therapy and 6 months after (*N =* 21). A median decline of − 18.2% (− 12.1 to − 20.6) was found prior to rituximab and a median improvement of 3.0% (4.6 to − 0.9) after rituximab therapy (*P =* 0.001)

The evolution of FVC and DLCO during the course of the study is given in Figs. 2 and 3. Prior to rituximab treatment, FVC showed a median decline of − 9.3% (− 5.2 to − 12.1) and 6 months after rituximab therapy FVC showed a median improvement of + 5.7% (− 4.2 to 9.2) (*P <*0.01). A median decline in DLCO of − 18.2% (− 12.1 to − 20.6) was found prior to rituximab and a median improvement of + 3.0% (− 0.9 to 4.6) after rituximab therapy (*P <* 0.001). There was no significant change in FVC and DLCO among different diagnosis groups.

### Biomarker changes

Biomarkers at baseline and following rituximab therapy are presented in Table 2. At baseline, CD19 B cells were within the normal range in all patients and decreased to non-measurable levels 2 to 4 weeks after rituximab treatment (*P* <0.01). There was no indication of B cell repopulation within the follow-up period. There were no differences among groups in B cell populations. CD4:CD8 ratio, CCL18, and sIL-2R did not change significantly during the study. The NR patients had higher PCT values than RSP patients (*P =* 0.03). No other biomarkers showed a significant difference among groups.

**Table 2:** Biomarkers before and after rituximab treatment (*N* = 20)

	Pre-rituximab	Post-rituximab	Significance
CD19 B cell 10*9/L (reference 0.10–0.60)	0.105 SD 0.1	0.0012 SD 0.0024	*P* ≤ 0.05
CD4:CD8 ratio (reference 1.0–3.5)	3.64 SD 4.2	3.22 SD 3.8	NS
CCL18 (reference 15–60 ng/ml)	133.9 SD 64.0	125.5 SD 56.3	NS
PCT (reference 0.10–0.49 ng/ml)	32.1 SD 19.1	40.0 SD 27.3	NS

*PCT*, procalcitonin; *CCL18*, chemokine ligand 18

None of the serum biomarkers correlated with DLCO changed after rituximab therapy. Post-rituximab CD4:CD8 ratios, sIL-2R values, and procalcitonin did show a moderate correlation with the change in FVC (*K =* 0.48, *P =* 0.043; *K =* 0.52, *P =* 0.02; *K* =− 0.50, *P =* 0.04, respectively).

### Chest imaging

After 6 months, half of the responders in RSP_improv_ showed improvement of ground glass and fine fibrosis on HRCT, and the other half showed no changes on HRCT. All RSP_stable_ patients showed no changes of ILD features on HRCT. In the NR group, worsening of ILD features on HRCT was found in 5 of the 6 patients. The 6th patient in this group showed an unchanged HRCT.

### Pulmonary function tests and [89Zr]-rituximab PET

[89Zr]rituximab target-to-background ratios demonstrated a moderate but significant positive relationship with both FVC change (*K* = 0.49, *P* = 0.029) and DLCO change (*K* = 0.56, *P* = 0.01). The [89Zr]-rituximab SUVmean of the lungs did not correlate with FVC and DLCO, *K =* 0.44 (*P* = 0.63) and *K* = − 0.29 (*P =* 0.24), respectively. SUVmax did not correlate with FVC and DLCO (*K =* 0.11, *P =* 0.64 and *K =* 0.31, *P =* 0.19, respectively).

### Chest imaging and [89Zr]-rituximab PET

**Fig. 4:**
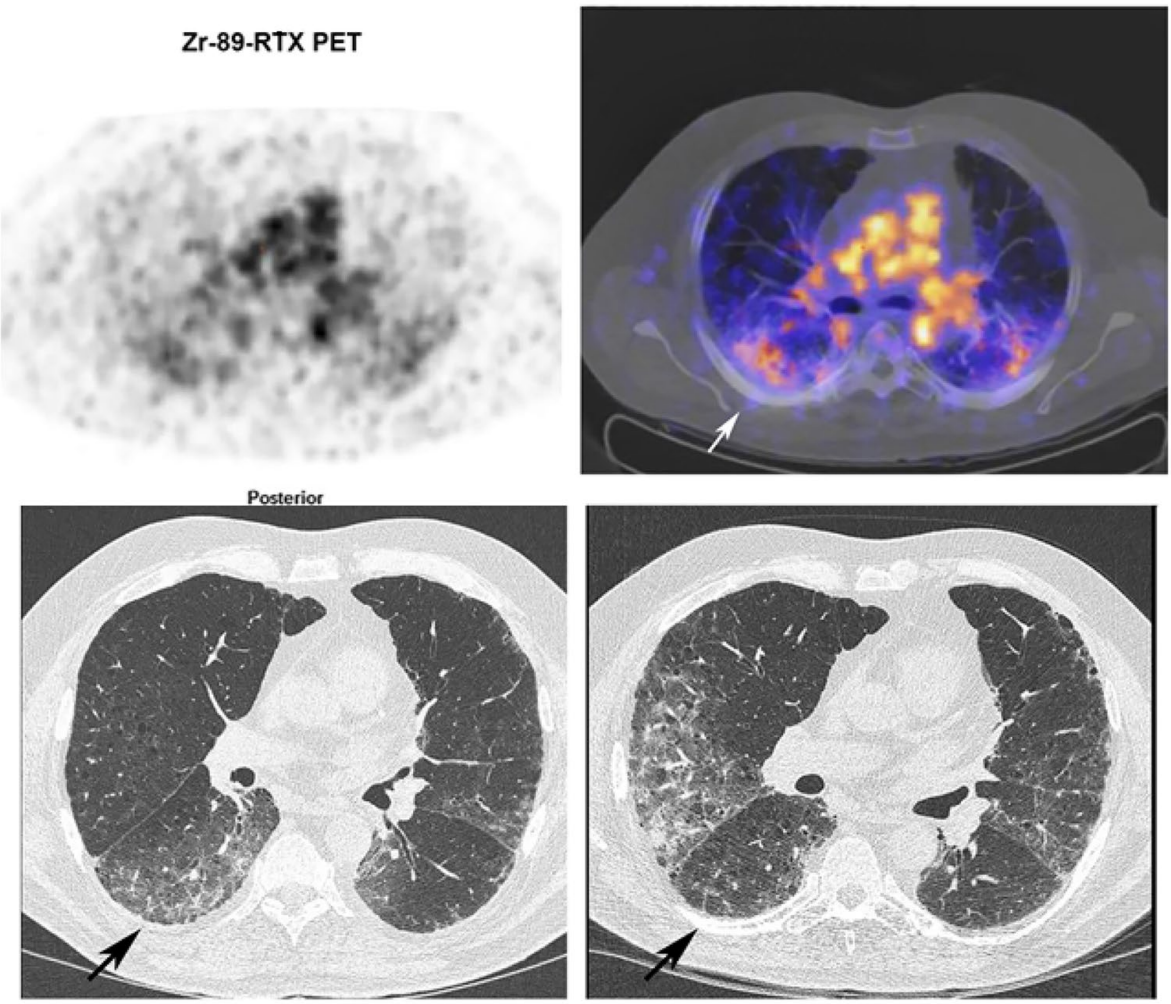
[89Zr]-rituximab PET/CT and HRCT of a 65-year-old patient with fibrotic non-specific interstitial pneumonia associated rheumatoid arthritis (case 3). [89Zr]-rituximab PET (top left) axial PET image (top right) fused [^89^Zr]-rituximab PET/CT. HRCT (bottom left) at baseline of PET and HRCT after one year (bottom right). Matching of the axial view between the PET/CT and HRCT is not exactly possible since the HRCT is performed with an inspiration command and the PET/CT is in the resting state. The [89Zr]-rituximab activity is more focused in the lower lobes. This central area on [89Zr]-rituximab correlates more with the HRCT ground glass; see, for example, the right lower lobe ground glass area. Interestingly the HRCT after one year post rituximab did not show any ground glass in this area. However, new ground glass areas emerged on the HRCT in the lower upper lobes. The patient remained stable in pulmonary function

Physiological [89Zr]-rituximab blood pool activity was observed (large vessels, cardiac chambers). Liver activity was stable in all patients, whereas spleen activity was variable. [89Zr]-rituximab expression in the lungs manifested as diffuse and patchy areas at different locations. Most active areas on [89Zr]-rituximab were not colocalized with HRCT abnormalities. However, in some cases with clinical response, [89Zr]-rituximab uptake correlated with HRCT findings (Fig. 4).

### Correlation between clinical response and [89Zr]-rituximab PET

**Fig. 5:**
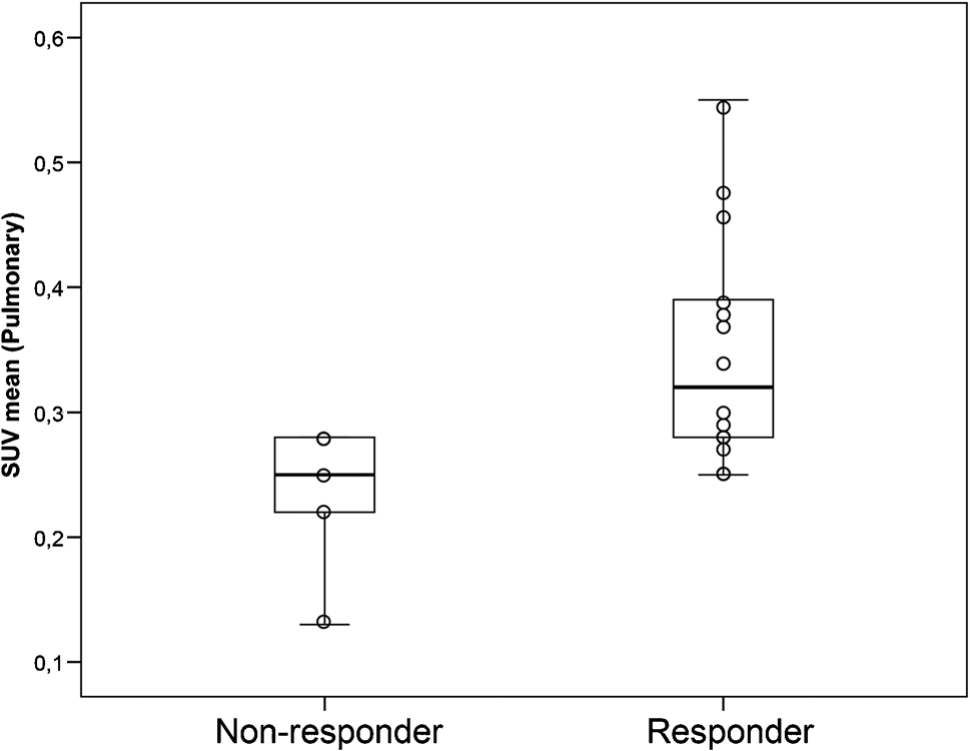
The mean SUV of pulmonary [89Zr]-rituximab uptake in non-responders (*n* = 5) ranged from a minimum of 0.13 to a maximum of 2.7, while in responders (*n* = 14), it ranged from a minimum of 0.23 to a maximum of 0.54 (*P* = 0.02). Patients with stability were also considered responders in this analysis

Figure 5 shows the different response categories and [89Zr]-rituximab activity in 19 patients. Six responders received [89Zr]-rituximab first, followed by therapeutic rituximab 6 days after, while eight received therapeutic rituximab first, followed by [89Zr]-rituximab within 4 h. The responders had higher absolute mean SUV compared with non-responders. One patient had missing data at day 3, and one outlier (NR case 5, with a SUV mean of 0.47) had a worsening clinical condition after a chest injury due to a fall and was therefore excluded from the final PET analysis. The SUV mean was significantly lower in NR (0.23 SD 0.06; *N* = 5) compared with RSP (0.35 SD 0.09; *N* = 14), *P* = 0.02.

## Discussion

This study investigated a novel non-invasive anti-CD20 imaging technique in patients with IMID-IP to assess the impact of anti-CD20 expression on subsequent rituximab therapy. While rituximab therapy resulted in complete depletion of B cells in all patients, the clinical outcome was still variable. This is the first study in IMID-IP to show that in vivo pulmonary CD20 expression differs significantly between responders and non-responders to rituximab therapy, as depicted by [89Zr]-rituximab PET/CT. The presence of CD20 cells in the lung was predictive of response in our study, and [89Zr]-rituximab PET/CT therefore has the potential to select patients who will benefit from rituximab therapy.

Recently, we have shown that anti-CD20 imaging PET can be used to assess the presence of CD20 in ILD in IMIDs [11]. Since there is immunohistochemical evidence of CD20 expression in subpleural and peribronchiolar lymphoid follicles in IMID-IP, in vivo molecular imaging of CD20 may become a useful biomarker for precision medicine [20]. In our prospective cohort of 21 IMID-IP patients, 72% showed a clinical response, consistent with results found in earlier retrospective studies [2, 17, 21–24]. Pulmonary anti-CD20 imaging at baseline correlated significantly with changes in FVC and DLCO at 6 months. We hypothesize that the improvement in pulmonary function correlates with a possible higher number of target CD20 cells in the lungs. However, histological confirmation was not possible, as biopsy was considered a life-threatening risk and therefore was not considered justified. Adequate specificity and reliability in anti-CD20 cell targeting using [89Zr]-rituximab PET have already been demonstrated in malignant lymphoma and rheumatoid arthritis by analyzing histology in mouse models and human application [13, 25–32].

Despite effective complete depletion of B cells 2 weeks after rituximab therapy, 28% of patients did not respond to therapy. In our previous study, we demonstrated that the recovery of “normal” B cells in the blood from the bone marrow was not a predictor of non-response to rituximab treatment [33]. Thus, it appears that in this refractory subset, factors other than peripheral B cell counts influence disease progression. In non-responders, other immune mechanisms might be active, and/or they might have more extensive irreversible pulmonary scarring. IMID-IP patients had several abnormalities in baseline biomarkers, but none of them showed an association with response outcome in this cohort, with the exception of procalcitonin (PCT). PCT was substantially higher on average in all patients, with baseline levels slightly higher in non-responders than in responders. However, during the course of rituximab therapy, the non-responders showed a significant increase in PCT levels compared with the responders. PCT therefore appears to be an interesting blood-based biomarker that warrants further investigation. Other explanations that could contribute to non-response include comorbidities and genetic factors.

The sites of CD20 accumulation in the lung are mostly diffuse with variable local areas of increased CD20 presence, such as subpleural regions and interstitial regions, but also some areas of fibrosis in agreement with histological findings [34]. CD20 expression has been found in several unpredictable locations in the lung, making [89Zr]-rituximab PET/CT a technique to identify clusters of CD20 aggregates when biopsy is considered clinically imperative. For example, we have demonstrated variable CD20 expression in the spleen that is unpredictably related to clinical outcome [33]. While this finding needs further investigation, it indicates that in vivo imaging of CD20 expression may provide better insight into the unpredictable migration and aggregation of CD20 cells and that this could have important clinical implications in undetermined cases, such as treatment decisions. Although anti-CD20 imaging is not a substitute for histology, identifying sites in the lung that are likely to express high levels of CD20 could be helpful, especially in the absence of other non-invasive alternatives.

The strengths of the study are the use of a robust clinical assessment and consistent treatment regimens. The diagnosis IMID-IP was made by an experienced multidisciplinary team of ILD specialists, a rheumatologist, and a thoracic radiologist. While IMIDs with ILD are heterogeneous diseases, treatment regimens are quite similar, especially in progressive disease. All patients enrolled in the study were refractory to maintenance doses of corticosteroids (prednisone) and immunosuppressants (azathioprine, cyclofosfamide, and/or methotrexate) for at least 6 months according to the study protocol. Within the study group, the treatment regimen was not changed except that rituximab was added. The corticosteroid maintenance dose was increased in 2 patients who did not respond.

Our study also has limitations. First, this study involves a relatively small number of patients in a single-center study with no control group. A small number of patients reduce the statistical power to detect small to moderate treatment effects and the ability to conduct credible subgroup analyses. Nevertheless, we found a significant treatment effect, indicating more than small to moderate benefit from treatment with rituximab. Furthermore, randomization to placebo in these vulnerable patients was considered unethical [35, 36]. In our single-arm study with historical controls, we only enrolled patients who were refractory to conventional immunosuppressants and required alternative treatment. Rapid progression of IMID-IP is associated with high mortality. A two-arm design with rituximab versus conventional immunosuppressants was also considered unethical because these patients were already unresponsive to these conventional immunosuppressants [37].

Another possible limitation of our study relates to the injection [89Zr]-rituximab in relation to prior exposure to therapeutic rituximab. The first 10 patients received therapeutic rituximab first, followed by [89Zr]-rituximab tracer within 4 h of therapeutic rituximab, as per a previously published pre-loading protocol for oncology patients [38]. However, in this rare pulmonary immunologic disease, there is no definitive answer as to the optimal protocol [39]. Although this time difference of 4 h to 6 days was within the physiologic distribution window (2 to 20 days), it is likely that the change in timing of [89Zr]-rituximab tracer injection had some small effect on whole-lung SUV values. As even in fast-growing tumors, the tumor uptake of [^89^Zr]-labeled immunotherapies is a slow process that takes days, and we hypothesize that this process is likely to be even slower in chronic inflammatory lung disease. According to the pharmacokinetics model, the lung has the slowest endosomal antibody uptake rate of any other organ in the body [40]. Therefore, in chronic pulmonary conditions, we expect that the impact of the different protocols should be less than is known from oncologic studies. Moreover, within the group of responding patients, the two injection protocols are evenly distributed. We also measured total lung volumes. The mean SUV of total lung volume remains largely unaffected by local (smaller) areas with (highly) increased SUVmax, as we have demonstrated this in our previous study[41]. Although our results need to be verified in future studies, we believe that changing the timing of tracer injection may have a potential benefit for the anti-CD20 PET image quality in inflammatory lung conditions by reduction of (partial) saturation by pre-loading.

The source of the radionuclide may also have an impact on outcomes. In a previous study, [68Ga]-pentixafor was used to analyze CXC chemokine receptor 4 (CXCR4) expression in fibrotic tissue [42]. The results correlated with the clinical outcome, but the PET imaging results could not be correlated with quantitative PFT values such as FVC. Gallium-68 provides a good signal-to-noise ratio but has a decay half-life of only 68 min, which may be too short for assessing slow organic processes such as monoclonal antibody targeting [25]. In contrast, zirconium-89, with a half-life of 3.27 days, matches longer circulating substances better than gallium-68 [42–44]. Although methods for imaging CD20 with gallium-68-labeled radiopharmaceuticals are known [45], we chose zirconium-89 for CD20 imaging because of its half-life matching pharmacokinetics of rituximab. However, the SUV mean values at day 6 were only about half those at day 3 and did not appear to be reliable due to poor signal-to-noise ratio. Day 6 results were therefore excluded from the final analysis. Finally, future studies could be improved by more sensitive CD20 imaging to achieve a better signal-to-noise ratio. The use of a highly sensitive digital PET scanner can also have a major impact on the detection of low signal effects in inflammatory pulmonary disease like IMID-IP.

In conclusion, this prospective phase 2 study in IMIDs with progressive life-threatening ILD demonstrates that 72% responded to rituximab treatment suggesting a significant benefit of this off-label rescue treatment option. In addition, we show that a novel radiolabeled anti-CD20 imaging technique might be useful as a non-invasive predictive biomarker. Our data showed a higher pulmonary CD20 presence in responders compared to non-responders when treated with rituximab and therefore warrant further study into this novel imaging technique.

## Supplementary Information

Below is the link to the electronic supplementary material.

Supplementary file1 (DOCX 25 KB)

## Data Availability

Not applicable.

## Declarations

### Disclaimer

The research council had no role in the design of the study, the collection and analysis of the data, or the preparation of the manuscript.
