# Supplementary material for: [89Zr]-immuno-PET prediction of response to rituximab treatment in patients with therapy refractory interstitial pneumonitis: a phase 2 trial
Source: Eur J Nucl Med Mol Imaging. 2023 Feb 24;50(7):1929–39. doi: 10.1007/s00259-023-06143-1 (PMC10199842; doi:10.1007/s00259-023-06143-1)
Supplement: Supplementary file 1 — Supplementary file1 (DOCX 25 KB) [file 259_2023_6143_MOESM1_ESM.docx]

SUPPLEMENT:

| Table s1. supplement study inclusion and exclusion criteria |
| --- |
|  |
| STUDY POPULATION |
|  |
| **Diagnosis of IMID-IP:** Immune-mediated inflammatory disease (IMID) and concurrent interstitial pneumonitis (IP) require a multidisciplinary approach to diagnosis. Patients are referred with a history IMID that includes pulmonary involvement in one of the following conditions: rheumatoid arthritis, polymyositis, dermatomyositis, mixed connective tissue disease, systemic sclerosis (systemic scleroderma), chronic extrinsic allergic alveolitis, or anti-synthetase syndrome. Diagnosis is made by a multidisciplinary committee using the most recent clinical guidelines for each subtype published by the American Thoracic Society (ATS), the European Respiratory Society (ERS), the Japanese Respiratory Society (JRS), and the Latin American Thoracic Society (ALAT). |
|  |
| Inclusion criteria |
| In order to be eligible to participate in the study, subjects must meet all of the following criteria: |
|  |
| • Age 18 - 70 years |
| • No previous therapy with rituximab |
| • At least 2 pulmonary function tests within past 6 months |
| • Diagnosis of co-existing IMID and a severe and / or progressive IP characterized by 3 out of 3 of the following items: |
| - Respiratory symptoms consistent with interstitial lung disease |
| - Diagnosis of usual interstitial pneumonia (UIP), non-specific interstitial pneumonia (NSIP), organizing pneumonia (OP) or a mixed form of UIP / NSIP / OP by either of the following: |
| - - - Open or video-assisted thoracic surgery (VATS) lung biopsy showing definite or probable UIP / NSIP / OP |
| - - - HRCT scan showing definite or probable UIP / NSIP / OP / mixed |
| - FVC <50% pred. and/or DLCO <40% pred., or worsening of lung function as demonstrated by any one of the following within the past year: |
| - - - > 10% decrease in FVC |
| - - - > 15% decrease in DLCO |
| • Therapy resistance to 1st (corticosteroids) and 2nd line therapy (cyclophosamide or azathioprine) |
|  |
| Exclusion criteria |
| A potential subject who meets any of the following criteria will be excluded from participation in this study: |
|  |
| • Residual volume >120% predicted at screening |
| • DLco <25% of predicted value at screening |
| • History of unstable or deteriorating cardiac or neurological disease |
| • Pregnancy or lactation |
| • Hematology lower than specified limits (leucocytes) |
| • Positive HIV, hepatitis B or C serology |
| • Pre-existing conditions which lead to a life expectancy of less than 6 months. |
| • Receipt of any vaccine, particularly live viral vaccines, within 4 weeks before first rituximab dose. |
| • Hypersensitivity for murine proteins |
| • NYHA IV stage heart failure |
|  |
| NOTE: Fever (> 37,9 °C) at presentation is reason to delay therapy by 1 week |
| Evidence of active infection is reason to postpone rituximab treatment until no further signs of active infection |
| Severe renal impairment is not a contraindication for rituximab therapy, however, if patients (might) require dialysis frequently they will be excluded from the study group |

| **Table s2. Description of all patients prior to treatment with rituximab** | | | | | | |
| --- | --- | --- | --- | --- | --- | --- |
| Patient number | Age | M/F | Diagnosis | Year of diagnosis | HRCT diagnosis | medication Pre-rituximab |
| 1 | 69 | M | RA | 2013 | UIP | azathioprine, prednisone |
| 2 | 67 | M | RA | 2010 | UIP | prednisone, cyclophosphamide, azathioprine |
| 3 | 65 | M | RA | 2013 | NSIP | cyclophosphamide, methotrexate |
| 4 | 44 | M | ASS | 2012 | UIP | prednisone, azathioprine |
| 5 | 57 | F | ASS | 2015 | fNSIP | prednisone, azathioprine |
| 6 | 69 | F | ASS | 2009 | fNSIP | prednisone, azathioprine |
| 7 | 59 | F | cEAA | 2015 | EAA | azathioprine cyclophosphamide |
| 8 | 63 | F | cEAA | 2015 | EAA | cyclophosphamide, prednisone |
| 9 | 57 | M | Scleroderma | 2014 | fNSIP | cyclophosphamide, azathioprine |
| 10 | 69 | F | Connective tissue disease | 2010 | fNSIP | cyclophosphamide, prednisone |
| 11 | 67 | F | RA | 2008 | UIP | prednisone, azathioprine |
| 12 | 63 | F | cEAA | 2007 | EAA | prednisone, azathioprine, mycophenolic acid |
| 13 | 71 | M | ASS | 2013 | fNSIP | prednisone, azathioprine, methotrexate |
| 14 | 30 | V | ASS | 2015 | fNSIP | prednisone, azathioprine, cyclophosphamide |
| 15 | 61 | V | ASS | 2012 | fNSIP with OP | prednisone, azathioprine |
| 16 | 70 | M | cEAA | 2014 | EAA | prednisone, azathioprine |
| 17 | 63 | V | cEAA | 2014 | EAA | prednisone, azathioprine |
| 18 | 68 | M | cEAA | 1991 | EAA | prednisone, cyclophosphamide, azathioprine |
| 19 | 55 | V | cEAA | 2006 | EAA | prednisone, azathioprine |
| 20 | 49 | V | Dermatomyositis | 2016 | fNSIP with OP | prednisone, cyclophosphamide |
| 21 | 51 | M | Scleroderma | 2014 | fNSIP | prednisone, cyclophosphamide, azathioprine |
| Rheumatoid arthritis (RA), antisynthetase syndrome (ASS), chronic extrinsic allergic alveolitis (cEAA), usual interstitial pneumonia (UIP), fibrotic (f) non-specific interstitial pneumonia (NSIP) organizing pneumonia (OP). | | | | | | |

|  | **Category** | **Category** | **Category** |
| --- | --- | --- | --- |
| **Patient** | **non-reponder** | **Stable** | **Improved** |
|  |  |  |  |
| Clinical Decline | 100% |  |  |
| Clinical Stable | 9% | 73% | 18% |
| Clinical Improved |  |  | 100% |
|  |  |  |  |
|  | **Category** | **Category** | **Category** |
| **Patient** | **non-reponder** | **Stable** | **Improved** |
|  |  |  |  |
| PFT Decline | 100% |  |  |
| PFT Stable | 33% | 67% |  |
| PFT Improved |  | 25% | 75% |
|  |  |  |  |
|  | **Category** | **Category** | **Category** |
| **Patient** | **non-reponder** | **Stable** | **Improved** |
|  |  |  |  |
| Imaging Decline | 100% |  |  |
| Imaging Stable | 15% | 62% | 23% |
| Imaging Improved |  |  | 100% |
| **Table s3**. Patient classification: The role of Clinical Response, PFTs, and Imaging | | | |
| Clinical response (including quality of life), pulmonary function test (PFT) status, and imaging are factors used to classify patients into "non-responder," "stable," or "improvement" categories. In the "stable" category, clinical and PFT stability are considered the most important factors. Imaging stability is also a factor, with a majority of patients in the "stable" category falling into the subcategory. In some cases, pulmonologists may classify patients as "stable" based on clinical and imaging parameters, even if PFTs show improvement. | | | |
